# Investigating the effect of prescribing status and patient characteristics on the therapeutic outcomes in patients with diabetes using a leftover drug adjustment protocol

**DOI:** 10.3389/jpps.2024.12886

**Published:** 2024-06-10

**Authors:** Toshiyuki Hirai, Shunsuke Hanaoka, Yuusuke Terakado, Toshiichi Seki, Fumiyuki Watanabe

**Affiliations:** ^1^ Department of Pharmacy, Hitachi, Ltd. Hitachinaka General Hospital, Ibaraki, Japan; ^2^ Laboratory of Pharmacotherapy, School of Pharmacy, Nihon University, Chiba, Japan; ^3^ Laboratory of Pharmacy Practice in Primary Care, School of Pharmacy, Nihon University, Chiba, Japan

**Keywords:** diabetes, medication adherence, pharmacy, leftover drug, diabetes treatment, multiple correspondence analysis, K-means cluster analysis

## Abstract

Treatment for diabetes includes anti-diabetic medication in addition to lifestyle improvements through diet and exercise. In Japan, protocol-based pharmacotherapy management allows drug treatment to be provided through cooperation between physicians and pharmacists, based on a protocol that is prepared and agreed upon in advance. However, there are no studies to clarify the relationship between patient characteristics and therapeutic effects after pharmacist intervention in protocol-based pharmacotherapy management for patients with diabetes. Therefore, this study aimed to use protocol-based reports from pharmacies to understand the status of outpatient diabetes medication compliance. We classified patients with diabetes on the basis of patient characteristics that can be collected in pharmacies and investigated the characteristics that impacted diabetes treatment. Patients were prescribed oral anti-diabetic drugs at outpatient clinics of Hitachinaka General Hospital, Hitachi, Ltd., from April 2016 to March 2021. Survey items included patient characteristics (sex, age, number of drugs used, observed number of years of anti-diabetic drug prescription, number of anti-diabetic drug prescription days, and presence or absence of leftover anti-diabetic drugs) and HbA1c levels. Graphical analyses indicated the relationship between each categorised patient characteristic using multiple correspondence analyses. Subsequently, the patients were clustered using K-means cluster analysis based on the coordinates obtained for each patient. Patient characteristics and HbA1c values were compared between the groups for each cluster. A total of 1,910 patients were included and classified into three clusters, with clusters 1, 2, and 3 containing 625, 703, and 582 patients, respectively. Patient characteristics strongly associated with Cluster 1 were ages between 65 and 74 years, use of three or more anti-diabetic drugs, use of 3 years or more of anti-diabetic drugs, and leftover anti-diabetic drugs. Furthermore, Cluster 1 had the highest number of patients with worsening HbA1c levels compared with other clusters. Using the leftover drug adjustment protocol, we clarified the patient characteristics that affected the treatment course. We anticipate that through targeted interventions in patients exhibiting these characteristics, we can identify those who are irresponsibly continuing with drug treatment, are not responding well to therapy, or both. This could substantially improve the efficacy of their anti-diabetic care.

## Introduction

The number of patients with diabetes worldwide is expected to exceed 600 million by 2030 [1]. In addition, >3.2 million people in Japan had diabetes in 2016 [2]. Approximately 10 million people are strongly suspected of having diabetes, and the possibility of diabetes cannot be ruled out in approximately 10 million people [3]. Diabetes can become a serious complication of long-term hyperglycaemia. Treatment for diabetes includes anti-diabetic drugs in addition to lifestyle improvements through diet and exercise [4]. Controlling blood glucose levels during diabetes treatment is important because it avoids complications [5–8]. Age, sex, educational background, and number of medications taken per day have been cited as patient characteristics associated with adherence to diabetes treatment [9–12].

In Japan, prescription forms include measures to be taken if a pharmacy checks for leftover drugs as part of the efforts to manage medication administration [13] and checking for leftover drugs at a pharmacy is included in medical fees [14]. A survey in the United States reported that the adherence rate to diabetes medication was 78.1% [10], while a survey in Japan reported that medication adherence among patients with diabetes was 79.6%, with approximately half reporting non-adherence [11]. In contrast, Collaborative Drug Therapy Management in the United States allows pharmacists to undertake professional responsibilities according to protocols under contracts between pharmacists and physicians [15]. In Japan, in protocol-based pharmacotherapy management, drug treatment can be provided in cooperation with physicians based on a protocol prepared and agreed upon in advance [16]. Using this protocol, Hitachi, Ltd. Hitachinaka General Hospital has already introduced a system (leftover drug adjustment protocol) for checking leftover drugs at pharmacies for outpatient prescriptions from our hospital. The leftover drug adjustment protocol allows pharmacists to adjust the number of prescription days based on the quantity of leftover drugs at the time of dispensing. After this adjustment, the number of adjusted prescription days is recorded on a specified form to monitor the status of leftover drugs and this information is reported to our hospital. The protocol also includes steps to identify the reason for the leftover medication on the form, to manage these leftover drugs, and to report to the hospital following the administration of the medication. To date, there have been surveys of medication adherence and blood glucose levels in Japanese patients with diabetes and evaluations of the content prepared using protocol-based pharmacotherapy management [17–21]. However, studies clarifying the relationship between patient characteristics and therapeutic effects after pharmacist intervention in protocol-based pharmacotherapy management for patients with diabetes are lacking.

Therefore, this study aimed to identify patient characteristics that may affect treatment efficacy by focusing on patient groups with low treatment efficacy based on multiple variables, such as the status of leftover anti-diabetic drugs. These data were obtained from pharmacy reports based on patient treatment protocols and electronic health records, and included basic patient characteristics that can be assessed in pharmacies.

In this study, we investigated various patient characteristics, including sex, age, number of drugs, number of days prescribed, duration of diabetes treatment, and the presence or absence of leftover anti-diabetic medications reported by pharmacies based on the protocol. When examining the relationship between each pair of multivariate variables or variables classified into multiple categories using pairwise strategies, we were able to observe significant associations. However, understanding how individual associations are constructed and grasping the overall picture remains challenging. Multiple correspondence analysis (MCA), a descriptive method, reduces dimensionality by mapping categorical variables and units of analysis to points in a low-dimensional space. MCA displays each variable or patient graphically, allowing visual exploration of relationships. The ability to visually discern relationships on a graph is a substantial advantage of MCA [22–25]. To elucidate the inter-variable linkage structure, we performed k-means cluster analysis on patient data that had undergone dimensionality reduction by MCA. This clustering approach facilitated the objective interpretation of patient characteristics that exhibit strong correlations within each cluster [24, 25]. Additionally, we compared patient characteristics and HbA1c transitions—an indicator of diabetes treatment efficacy—across clusters using multiple comparisons and residual analysis to identify patient groups with low therapeutic effects.

## Materials and methods

### Study design

This was a single-centre, retrospective, observational cohort study. This study complied with the standards of the Declaration of Helsinki and current Ethical Guidelines. The design and methodology, which include the opt-out method of consent available to all patients, were approved by the Ethics Committees of Hitachinaka General Hospital, Hitachi, Ltd. (approval number: 16-010) and Nihon University School of Pharmacy (approval number: 21-008). This was a retrospective observational study using electronic medical records, and there was no interference with the patients.

### Study population

This study included 2,367 outpatients who were prescribed anti-diabetic drugs at our hospital for 5 years from 1 April 2016 to 31 March 2021. Two exclusion criteria were applied. First, 355 patients prescribed insulin injections and 116 patients prescribed GLP-1 analogue injections were excluded because residual medication adjustment was not performed unless one injection remained. Second, 61 patients without a history of HbA1c measurement were excluded because diabetes treatment could not be evaluated if their HbA1c levels were not measured. Ultimately, 1,910 patients were included in the study.

### Study items

Patient information was retrospectively obtained from electronic medical records. Study items included patient characteristics such as sex, age, number of prescription drugs, number of prescription anti-diabetic drugs, number of prescription days of anti-diabetic drugs, number of years of anti-diabetic drugs during the study period, and the presence or absence of adjustment of leftover anti-diabetic drugs. Furthermore, HbA1c was investigated as an index of the effect of diabetes treatment.

#### Age

Age was defined as the age at the first visit during the study period. In addition to comparing age (years), age was categorised as under 45 years (<45 years), over 45 years and under 54 years (45–54 years), over 55 years and under 64 years (55–64 years), over 65 years and under 74 years (65–74 years), over 75 years and under 89 years (75–89 years), and over 90 years (≥90 years) [Age (group): <45 years, 45–54 years, 55–64 years, 65–74 years, 75–89 years, and ≥90 years]. Although the WHO defines people aged ≥65 years as elderly, those aged ≥65 years were classified as 65–74 years (pre-old), 75–89 years (old), and ≥90 years (oldest old, super old), based on the recommendations of the Japan Gerontological Society and the Japan Geriatrics Society [26], which study the ageing situation in developed countries.

#### Number of prescribed drugs (all drug counts)

The number of prescribed drugs was defined as the number of drugs prescribed in the outpatient clinic of our hospital. Therefore, the number prescribed in other hospitals was unknown and not counted. Additionally, the number of prescribed drugs was classified into two groups [all drug counts (group): <5 and ≥5] based on past reports of polypharmacy [27].

#### Details of prescription of anti-diabetic drugs [diabetes mellitus (DM) drug counts, days of DM drug prescription, DM drug prescription duration]

The number of prescribed anti-diabetic drugs was defined as the maximum number of drugs prescribed during the study period. The number of prescribed drugs was classified into three categories [DM drug counts (group): 1, 2, or ≥3]. The number of prescription days (d) for anti-diabetic drugs is the maximum number of days per prescription. Prescription days were categorised as <30 d, ≥30 d but <60 d, 60 d or more [days of DM drug prescription (group): <30 d, 30–59 d, and ≥60 d]. Number of years of anti-diabetic drugs during the study period (years) was defined as the number of continuous years during the study period. Furthermore, prescription years were classified into four categories [DM drug prescription duration (group): <1 year, 1–2 years, 2–3 years, and ≥3 years].

#### Status of leftover diabetes drugs (leftover DM drugs)

The status of leftover drugs was investigated using reports from pharmacies, according to the leftover drug adjustment protocol. According to this protocol, when a pharmacist confirms that a leftover drug is present at the time of dispensing, a special form for reporting leftover drug status is used to adjust the number of prescription days with the leftover drug. The number of prescription days adjusted for the leftover drugs is subsequently recorded on the form. The protocol also includes selecting the reason in the form for the leftover drugs, for handling the leftover medicine, and for reporting it to the hospital after it has been administered. Additionally, if anti-diabetic drugs were included in the report based on the leftover drug adjustment protocol, the status of leftover anti-diabetic drugs was defined as (+), or were not included, (−) (leftover DM drugs: + and −).

#### HbA1c classification

We investigated HbA1c levels on the initial and final days of testing during the study period (initial or final HbA1c value recorded). Each HbA1c level was divided into four ranges with reference to the glycaemic control target of the Diabetes Clinical Practice Guidelines 2019 [28]. Range 1 was HbA1c <6.0%, Range 2 was HbA1c 6.0% to <7.0%, Range 3 was HbA1c 7.0% to <8.0%, and Range 4 was HbA1c ≥8.0% [initial or final HbA1c value recorded (all groups): 1, 2, 3, and 4]. Additionally, since the goal of preventing complications in diabetes treatment guidelines is HbA1c <7.0%, HbA1c was divided into <7.0% and ≥7.0% [initial or final HbA1c value recorded (group): <7% and ≥7%]. Furthermore, the variation in HbA1c ranges was calculated by taking the difference in the number of ranges between the initial HbA1c and final HbA1c recorded and setting it to +1, +2, and +3 as the number of ranges increased, with 0 as no change in interval for both intervals; and −1, −2, and −3 as the number of ranges decreased [final HbA1c–initial HbA1c (all group): −3, −2, −1, 0, +1, +2, and +3]. The primary outcome of diabetes treatment in this study was defined as not up when the variation of HbA1c ranges was 0, −1, −2, or −3 and up when it was +1, +2, or +3 [final HbA1c–initial HbA1c (group): not up and up].

### Statistical analysis

Statistical analyses were performed using JMP Pro 17 (SAS Institute Inc., Cary, NC, United States) and R Ver 4.3.2 [R Core Team (2023). R: A Language and Environment for Statistical Computing. R Foundation for Statistical Computing, Vienna, Austria^1^].

#### Patient characteristics, including HbA1c values for all patients

Patient characteristics and HbA1c values were expressed as medians (interquartile ranges) for continuous variables and as numerical values (%) for categorical variables.

#### Multiple correspondence analysis (MCA) and K-means cluster analysis

Multivariate analysis was performed using MCA to determine the relationship between patient characteristics (MCA; JMP Pro 17). MCA is an extension of correspondence analysis when multiple variables are considered and is a method of analysing categorical or categorised data and presenting the results in a graph (map). The information described in each dimension was evaluated using the Greenacre inertia adjustment and the categorical variables were plotted on the two dimensions with the highest inertia [22]. Additionally, each patient was plotted on the same map to demonstrate their relationship with each categorical variable [23]. The categories of basic information on patient characteristics input into the MCA were as follows: Basic information on patient characteristics: Sex (female and male), Age (group: <45 years, 45–54 years, 55–64 years, 65–74 years, 75–89 years, and ≥90 years), All drug counts (<5 and ≥5), DM drug counts (1, 2, and ≥3), days of DM drug prescription (<30 d, 30–59 d, and ≥60 d), DM drug prescription duration (<1 year, 1–2 years, 2–3 years, and ≥3 years), and leftover DM drugs (+ and −).

K-means cluster analysis was necessary to objectively classify patients based on each category of patient characteristics. This non-hierarchical cluster analysis identifies mutually exclusive clusters by calculating the quadratic Euclidean distance (similarity coefficient) for each patient on the map. The coordinates (object scores) of patient dimensions 1 and 2 calculated using MCA were input into the K-means cluster analysis, and the patients were clustered. These object scores were derived from the quantification of all categories of patient characteristics, which were treated as qualitative variables defining the individual profile. As composite scores, they maintained the multidimensionality of the input when cluster analysis was performed. K-means cluster analysis constructed a predetermined number of patient clusters using an iterative algorithm that partitioned the observations. This method organised patients into clusters to minimise the distances between each patient’s object score and the cluster centroids. Specifying the number of clusters was a prerequisite for the analysis [24, 25]. To determine the optimal number of clusters, the cubic clustering criterion (CCC) was calculated using statistical software. To ensure the interpretability of the results after clustering, we set the maximum number of clusters to 22, corresponding to the total number of patient characteristic categories used in the MCA. We then calculated the CCC for cluster solutions ranging from 1 to 22. Among the computed CCC values, we selected the number of clusters associated with the highest CCC as our final choice [29, 30]. After clustering the patients by specifying the number of clusters, the density ellipse (*α* = 95%) of each cluster of patients was shown and superimposed on the MCA map to indicate the overlap between clusters (K-means cluster analysis; JMP Pro 17).

#### Comparison of patient characteristics and HbA1c for each cluster

For basic information on patient characteristics and HbA1c levels, continuous variables were expressed as medians [interquartile ranges], and categorical variables were expressed as numbers (%). For continuous variables, we used the Steel–Dwass test to perform multiple comparisons between clusters (Steel–Dwass test; JMP Pro 17). For categorical variables, the chi-squared test was conducted for multiple comparisons between clusters. Additionally, cross-tabulation residual analysis was used to identify which groups differed within each item of patient characteristics and HbA1c values. We applied the Benjamini and Hochberg (BH) method to adjust *p*-values in multiple analyses of categorical variables and residual analysis (chi-squared test and residual analysis with BH adjustment; R Ver 4.3.2). Statistical significance was set at *p* < 0.05.

## Results

### Patient characteristics

Table 1 presents the characteristics of the target patients. The median age of the patients was 72.0 [64–79] years, with 35.4% of the total being women. Patients who were prescribed ≥5 drugs were 57.4%, and 59.6% (two drugs: 30.2%, three or more drugs: 29.4%) of patients were prescribed two or more anti-diabetic drugs. The median number of prescription days per prescription was 63.0 [49.0–77.0] d, and the median observed prescription duration was 2.0 [0.6–4.0] years. Approximately 20.3% of patients had leftover diabetes medication.

**TABLE 1:** Characteristics of target patients.

Patient characteristics	n or Median	Ratio (%) or [Interquartile range]
Overall	1910	(100)
Sex		
Female	676	(35.4)
Male	1,234	(64.6)
Age (years)	72	[64.0–79.0]
Age (group)		
<45 years	74	(3.9)
45–54 years	142	(7.4)
55–64 years	290	(15.2)
65–74 years	633	(33.1)
75–89 years	731	(38.3)
≥90 years	40	(2.1)
All drug counts	5.5	[3.7–7.8]
All drug counts (group)		
<5	813	(42.6)
≥5	1,097	(57.4)
DM drug counts	2.0	[1.0–3.0]
DM drug counts (group)		
1	773	(40.5)
2	576	(30.2)
≥3	561	(29.4)
DM drug prescription days (d)	63.0	[49.0–77.0]
Days of DM drug prescription (group)		
<30 d	198	(10.4)
30–59 d	445	(23.3)
≥60 d	1,267	(66.3)
DM drug prescription duration (years)	2.0	[0.6–4.0]
DM drug prescription duration (group)		
<1 year	640	(33.5)
1–2 years	314	(16.4)
2–3 years	349	(18.3)
≥3 years	607	(31.8)
Leftover DM drugs (group)		
−	1,522	(79.7)
+	388	(20.3)

The term “All drug counts” refers to the total number of drugs prescribed in the hospital’s outpatient clinic. “DM drug counts” indicates the maximum number of diabetes mellitus (DM) drugs concurrently prescribed and taken in the outpatient clinic. “Days of DM drug prescription (d)” represents the maximum number of days for a single prescription of DM drugs. “DM drug prescription duration (years)” denotes the continuous prescription duration in years during the study. The presence or absence of leftover diabetes drugs, based on the leftover drug adjustment protocol, is indicated by “leftover DM drugs: +/−.” Categorical variables are presented as numbers (%), and continuous variables are expressed as medians [interquartile range]. DM, diabetes mellitus.

### HbA1c

Table 2 depicts the changes in HbA1c in the target patients during the study period; 40.8% of the patients had an initial HbA1c ≥7%, and 41.8% had a final HbA1c ≥7%. During the study period, 23.9% of the patients had an increased HbA1c category.

**TABLE 2:** HbA1c values in all patients.

HbA1c Values	n or Median	Ratio (%) or [Interquartile range]
Overall	1910	(100)
Initial HbA1c recorded	6.8	[6.3–7.4]
Initial HbA1c recorded (all groups)		
1	202	(10.6)
2	929	(48.6)
3	470	(24.6)
4	309	(16.2)
Initial HbA1c recorded (all groups)		
<7%	1,131	(59.2)
≥7%	779	(40.8)
Final HbA1c recorded	6.8	[6.3–7.4]
Final HbA1c recorded (all groups)		
1	254	(13.3)
2	857	(44.9)
3	539	(28.2)
4	260	(13.6)
Final HbA1c recorded (group)		
<7%	1,111	(58.2)
≥7%	799	(41.8)
Final HbA1c–initial HbA1c	0	[−0.5–0.4]
Final HbA1c–initial HbA1c (all groups)		
−3	17	(0.9)
−2	117	(6.1)
−1	341	(17.9)
0	979	(51.3)
+1	372	(19.5)
+2	79	(4.1)
+3	5	(0.3)
Final HbA1c–initial HbA1c (group)		
Not up	1,454	(76.1)
Up	456	(23.9)

“Initial HbA1c recorded” represents the Haemoglobin A1c (HbA1c) level measured at the initial examination in the study period. “final HbA1c recorded” refers to the HbA1c level at the final examination in the same period. Each HbA1c level was divided into four ranges. Range 1 was HbA1c <6.0%, Range 2 was HbA1c 6.0% to <7.0%, Range 3 was HbA1c 7.0% to <8.0%, and Range 4 was HbA1c ≥8.0% [initial or final HbA1c value recorded (all groups): 1, 2, 3, and 4]. Additionally, HbA1c was divided into <7.0% and ≥7.0% [initial or final HbA1c value recorded (group): <7% and ≥7%]. “final HbA1c−initial HbA1c” is the variation in HbA1c ranges calculated by taking the difference in the number of ranges between the initial HbA1c and final HbA1c recorded, which was set to +1, +2, and +3 as the number of range increased, with 0 as no change in range for both ranges; and −1, −2, and −3 as the number of ranges decreased [final HbA1c–initial HbA1c (all group): −3, −2, −1, 0, +1, +2, and +3]. “Not up” represents the outcome when the final HbA1c–initial HbA1c (all group) was 0, −1, −2, or −3; and “up” represents when it was +1, +2, or +3 [final HbA1c–initial HbA1c (group): not up and up]. Categorical variables are presented as numbers (%), and continuous variables are expressed as medians [interquartile range]. HbA1c, Haemoglobin A1c.

### Results of MCA and K-mean cluster analysis

**FIGURE 1:**
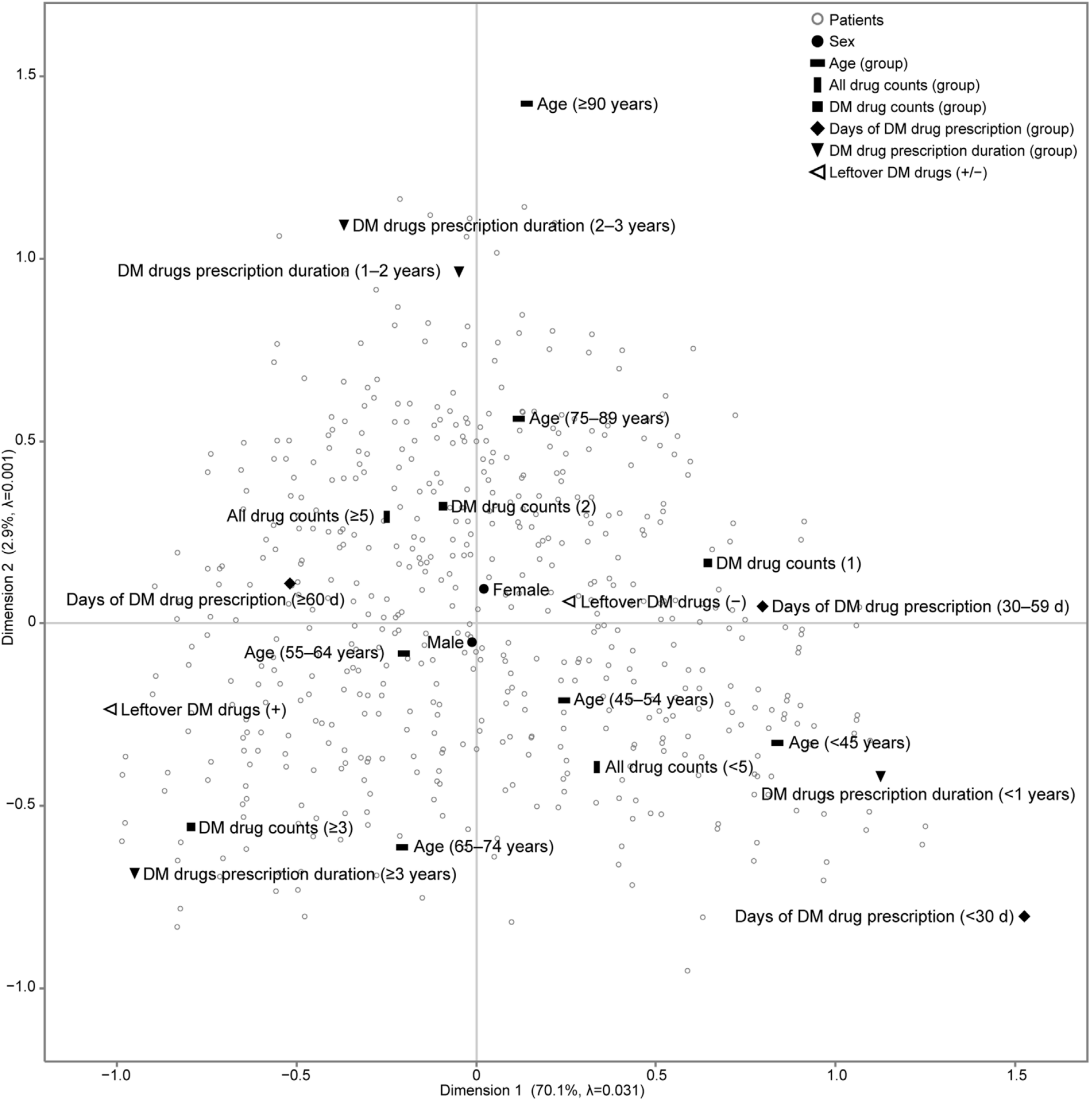
Results of multiple correspondence analysis (MCA) of patients with diabetes mellitus in the outpatient clinic of the hospital. We analysed a Burt matrix, which is a comprehensive multidimensional contingency table including all variables, to conduct the MCA. The placement of categorical variables on the map, such as patient characteristics and the status of leftover diabetes medication, illustrates the relationships between these variables. Additionally, each patient is plotted on the same map to show their relationship with each categorical variable. The number of all drugs prescribed is the count of all drugs prescribed in the outpatient clinic of the hospital. DM drug counts denotes the maximum number of DM drugs prescribed and taken simultaneously in the outpatient clinic of the hospital. Days of DM drug prescription (d) denotes the maximum number of days of a single prescription. DM drug prescription duration (years) denotes the number of years of continuous prescriptions during the study period. adjusted λ, Greenacre’s adjusted inertia; DM, diabetes mellitus.

We analysed a Burt matrix, which is a comprehensive multidimensional contingency table that includes all variables (data not shown), to perform MCA [23]. The first two dimensions accounted for 73.0% (Dimension 1: 70.1%; Dimension 2: 2.9%) of the Greenacre-adjusted inertia in the first two dimensions in Figure 1.

**FIGURE 2:**
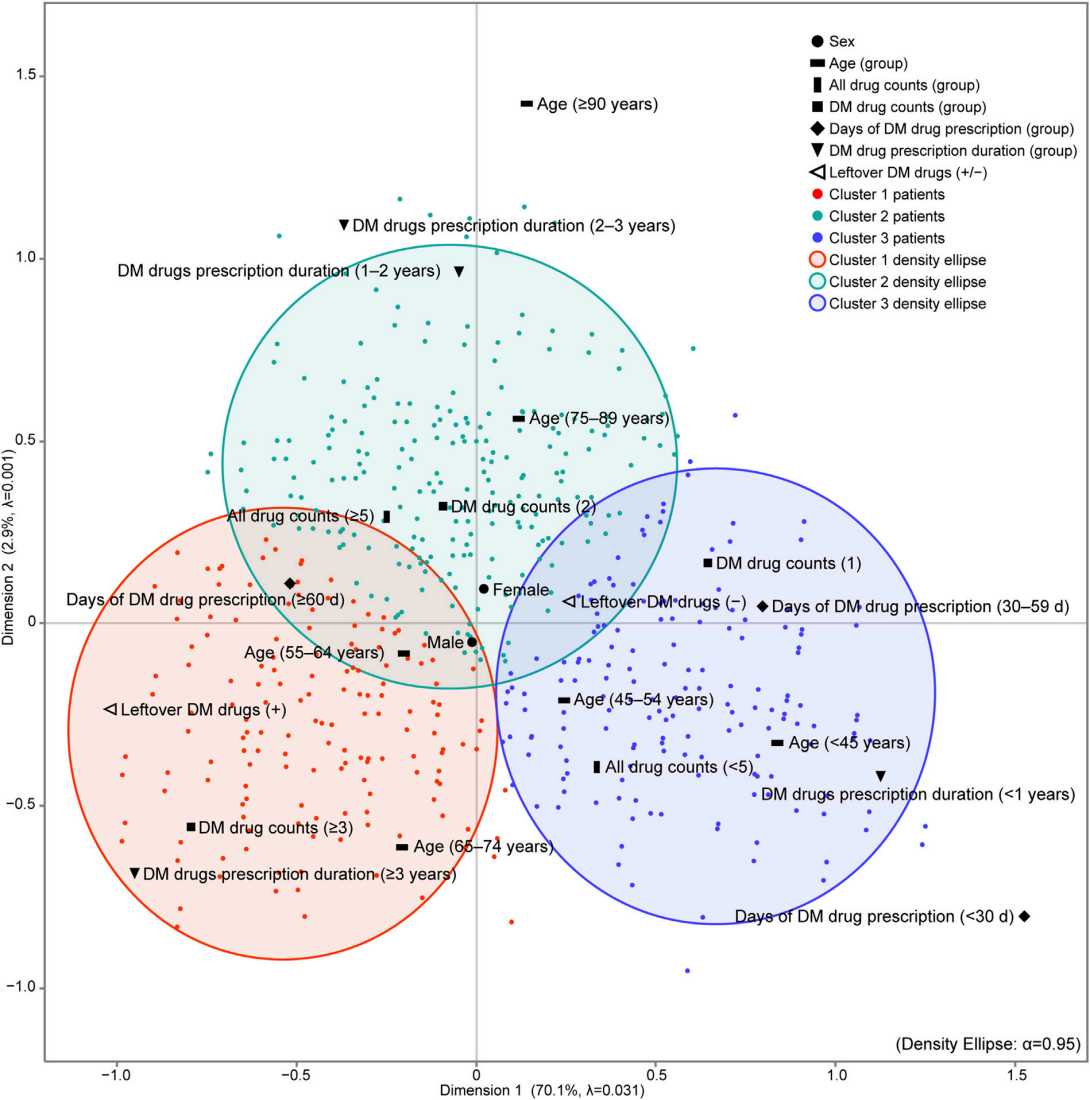
Results of MCA and K-means cluster analysis of patients with diabetes mellitus in the outpatient clinic. K-means cluster analysis was performed using the object scores of each patient for dimensions 1 and 2 to which each category was assigned. The maximum number of clusters was set to 22, which is the total number of categories of patient characteristics input into the MCA. After comparing the CCC for 1–22 clusters, three clusters emerged with the highest CCC peaks, with a CCC value of 11.9. The iterative algorithms were executed six times to minimise the distance between the centroids of the three clusters and the object scores of each patient, ensuring accurate cluster assignment of patients. For each cluster identified, we calculated a 95% probability ellipse that delineated the distribution range of the patients, which was then overlaid on the MCA map. The number of all drugs prescribed is the count of all drugs prescribed in the outpatient clinic of the hospital. DM drug counts denotes the maximum number of DM drugs prescribed and taken simultaneously in the outpatient clinic of the hospital. Days of DM drug prescription (d) denotes the maximum number of days of a single prescription. DM drug prescription duration (years) denotes the number of years of continuous prescriptions during the study period. adjusted λ, Greenacre’s adjusted inertia; DM, diabetes mellitus.

K-means cluster analysis was performed using each patient’s object scores (data not shown) for Dimensions 1 and 2, to which each category was assigned. After comparing the CCC for 1 to 22 clusters, three clusters emerged with the highest CCC peaks, with a CCC value of 11.9. For sensitivity analysis, K-means cluster analysis was performed on the outcome of MCA excluding sex, which is the patient characteristic closest to the origin on the map, and three cluster numbers were identified (CCC = 12.9). The iterative algorithms were executed six times to minimise the distance between the centroids of the three clusters and the object scores of each patient, ensuring accurate cluster assignment of patients. For each identified cluster, we calculated a 95% probability ellipse that delineated the distribution range of the patients, which was then overlaid on the MCA map (Figure 2). The patient characteristic categories encompassed by the probability ellipse are indicative of the characteristics most strongly associated with the patients constituting the cluster.

Cluster 1 was characterised by a Leftover DM drugs (+), indicating that these individuals had a higher count of DM drugs (≥3), a longer prescription duration (≥3 years), and were within the age range of 65–74 years compared to the other clusters. In contrast, Cluster 2 and Cluster 3 did not have a specific characterisation for leftover DM drugs.

### Results of multiple comparisons of patient characteristics across clusters, and residual analysis in cross-tabulation of patient characteristics items

Tables 3, 4 present a comprehensive analysis of patient characteristics across three distinct clusters. Table 3 presents the results of multiple comparisons of patient characteristics for each cluster. We applied the Steel–Dwass test for continuous variables and the chi-squared test with BH adjustment for categorical variables. Table 4 shows the results of the residual analysis with BH adjustment for the cross-tabulation of each patient characteristic category and cluster.

**TABLE 3:** Results of multiple comparisons for each cluster of patient characteristics.

Patient characteristics	Cluster 1 *n* = 625		Cluster 2 *n* = 703		Cluster 3 *n* = 582		Significantly different cluster combinations (*p* < 0.05)
	*n* or Median	Ratio (%) or [Interquartile range]	*n* or Median	Ratio (%) or [Interquartile range]	*n* or Median	Ratio (%) or [Interquartile range]	
Sex							N. A
Female	220	(35.2)	255	(36.3)	201	(34.5)	
Male	405	(64.8)	448	(63.7)	381	(65.5)	
Age (years)	70	[63.0–74.0]	76	[66.0–81.0]	71	[62.0–78.3]	1 vs. 2, 1 vs. 3, 2 vs. 3
Age (group)							1 vs. 2, 1 vs. 3, 2 vs. 3
<45 years	13	(2.1)	18	(2.6)	43	(7.4)	
45–54 years	43	(6.9)	45	(6.4)	54	(9.3)	
55–64 years	119	(19.0)	95	(13.5)	76	(13.1)	
65–74 years	306	(49.0)	141	(20.1)	186	(32.0)	
75–89 years	142	(22.7)	373	(53.1)	216	(37.1)	
≥90 years	2	(0.3)	31	(4.4)	7	(1.2)	
All drug counts	5.8	[4.0–7.9]	6.3	[4.3–8.5]	4.1	[2.6–6.4]	1 vs. 2, 1 vs. 3, 2 vs. 3
All drug counts (group)							1 vs. 2, 1 vs. 3, 2 vs. 3
<5	239	(38.2)	222	(31.6)	352	(60.5)	
≥5	386	(61.8)	481	(68.4)	230	(39.5)	
DM drug counts	3.0	[2.0–3.0]	2.0	[1.0–2.0]	1.0	[1.0–2.0]	1 vs. 2, 1 vs. 3, 2 vs. 3
DM drug count (group)							1 vs. 2, 1 vs. 3, 2 vs. 3
1	75	(12.0)	328	(46.7)	370	(63.6)	
2	159	(25.4)	272	(38.7)	145	(24.9)	
≥3	391	(62.6)	103	(14.7)	67	(11.5)	
Days of DM drug prescription (d)	70.0	[63.0–84.0]	70.0	[63.0–84.0]	35.0	[28.0–56.0]	1 vs. 2, 1 vs. 3, 2 vs. 3
Days of DM drug prescription (group)							1 vs. 2, 1 vs. 3, 2 vs. 3
<30 d	2	(0.3)	3	(0.4)	193	(33.2)	
30–59 d	38	(6.1)	119	(16.9)	288	(49.5)	
≥60 d	585	(93.6)	581	(82.6)	101	(17.4)	
DM drug prescription duration (years)	4.7	[3.6–5.0]	2.1	[1.4–2.6]	0.3	[0.1–0.6]	1 vs. 2, 1 vs. 3, 2 vs. 3
DM drug prescription duration (group)							1 vs. 2, 1 vs. 3, 2 vs. 3
<1 year	24	(3.8)	58	(8.3)	558	(95.9)	
1–2 years	29	(4.6)	269	(38.3)	16	(2.7)	
2–3 years	37	(5.9)	309	(44.0)	3	(0.5)	
≥3 years	535	(85.6)	67	(9.5)	5	(0.9)	
Leftover DM drugs							1 vs. 2, 1 vs. 3, 2 vs. 3
−	365	(58.4)	595	(84.6)	562	(96.6)	
+	260	(41.6)	108	(15.4)	20	(3.4)	

All drug counts’ refers to the total number of medications prescribed in the hospital’s outpatient clinic. “DM drugs count” indicates the highest number of DM medications prescribed and administered concurrently in the outpatient clinic. “Days of DM drug prescription (d)” represents the maximum duration of a single DM drug prescription in days. “DM drug prescription duration (years)” is the total number of years of continuous DM drug prescription during the study. If antidiabetic drugs were accounted for in the report based on the leftover drug adjustment protocol, this is indicated by DM drugs Leftover: “+” for included, or “−” for not included. For categorical variables, we performed the chi-squared test with a Benjamini and Hochberg (BH) adjustment for multiple comparisons, and the Steel–Dwass test for continuous variables between clusters. Statistical significance was set at *p* < 0.05. The results of multiple comparisons are denoted by “vs.” for the two significantly different clusters (*p* < 0.05). Categorical variables are presented as numbers (%), and continuous variables as medians [interquartile range]. DM, diabetes mellitus; N.A., not applicable.

**TABLE 4:** Results of residual analysis for each cluster of patient characteristics.

Patient characteristics	Cluster 1 *n* = 625			Cluster 2 *n* = 703			Cluster 3 *n* = 582				Adjusted residuals	Adjusted *p*
	Ratio (%)	Adjusted residuals	Adjusted *p*	Ratio (%)	Adjusted residuals	Adjusted *p*	Ratio (%)	Adjusted residuals	Adjusted *p*			
**Sex**											>0	<0.05
Female	35.2	−0.1	0.902	36.3	0.6	0.902	34.5	−0.5	0.902		>0	<0.1
Male	64.8	0.1	0.902	63.7	−0.6	0.902	65.5	0.5	0.902			≥0.1
**Age (group)**											<0	<0.1
<45 years	2.1	−2.8	0.009	2.6	−2.3	0.042	7.4	5.3	<0.001		<0	<0.05
45–54 years	6.9	−0.6	0.519	6.4	−1.3	0.227	9.3	2.0	0.069			
55–64 years	19.0	3.3	0.002	13.5	−1.6	0.155	13.1	−1.7	0.120			
65–74 years	49.0	10.2	<0.001	20.1	−9.3	<0.001	32.0	−0.7	0.519			
75–89 years	22.7	−9.8	<0.001	53.1	10.1	<0.001	37.1	−0.7	0.519			
≥90 years	0.3	−3.8	<0.001	4.4	5.4	<0.001	1.2	−1.8	0.108			
**All drug counts (group)**												
<5	38.2	−2.7	0.008	31.6	−7.4	<0.001	60.5	10.5	<0.001			
≥5	61.8	2.7	0.008	68.4	7.4	<0.001	39.5	−10.5	<0.001			
**DM drug counts (group)**												
1	12.0	−17.7	<0.001	46.7	4.2	<0.001	63.6	13.6	<0.001			
2	25.4	−3.1	0.002	38.7	6.2	<0.001	24.9	−3.3	0.001			
≥3	62.6	22.2	<0.001	14.7	−10.8	<0.001	11.5	−11.3	<0.001			
**Days of DM drug prescription (group)**												
<30 d	0.3	−10.1	<0.001	0.4	−10.9	<0.001	33.2	21.6	<0.001			
30–59 d	6.1	−12.4	<0.001	16.9	−5.0	<0.001	49.5	17.9	<0.001			
≥60 d	93.6	17.6	<0.001	82.7	11.5	<0.001	17.4	−30.0	<0.001			
**DM drugs prescription duration (group)**												
<1 year	3.8	−19.2	<0.001	8.3	−17.9	<0.001	95.9	38.2	<0.001			
1–2 years	4.6	−9.7	<0.001	38.3	19.6	<0.001	2.8	−10.7	<0.001			
2–3 years	5.9	−9.7	<0.001	44.0	22.2	<0.001	0.5	−13.3	<0.001			
≥3 years	85.6	35.2	<0.001	9.5	−15.9	<0.001	0.9	−19.2	<0.001			
**Leftover DM drugs**												
−	58.4	−16.1	<0.001	84.6	4.1	<0.001	96.6	12.1	<0.001			
+	41.6	16.1	<0.001	15.4	−4.1	<0.001	3.4	−12.1	<0.001			

All drug counts’ refers to the total number of medications prescribed in the hospital’s outpatient clinic. “DM drugs count” represents the highest number of diabetes mellitus (DM) medications concurrently prescribed and taken in the clinic. “Days of DM drug prescription days (d)” indicates the maximum number of days for a single DM drug prescription. “DM drug prescription duration (years)” measures the years of continuous DM drug prescriptions during the study. For diabetes drugs, their inclusion based on the leftover drug adjustment protocol is marked as DM drugs Leftover: “+” for included, and “−” for not included. Residual analysis was conducted for each cell, with a Benjamini and Hochberg (BH) adjustment applied to the *p*-values. Red cells signify adjusted residuals greater than 0 with adjusted *p*-values less than 0.05, indicating a significantly higher cell frequency than expected. Light red cells, with adjusted residuals greater than 0 and adjusted *p*-values less than 0.1, suggest a trend towards higher frequency, but not significantly. Conversely, blue cells (adjusted residuals less than 0, adjusted *p*-values less than 0.05) denote significantly lower frequencies, and light blue cells (adjusted residuals less than 0, adjusted *p*-values less than 0.1) indicate a trend towards lower frequencies, although not significant. DM, diabetes mellitus.

The patient characteristics represented on the map in Figure 2 by the MCA and K-cluster analysis are unique to each cluster. Also in the hypothesis testing results, these characteristics had the highest values in their respective clusters (Table 3) and significantly more than the expected frequency (Table 4). Notably, Cluster 1 had the highest proportion of patients aged 55–74 (especially between the ages 65–74), the highest count and prescription days of DM drugs, the longest prescription duration, and the highest proportion of patients with leftover medication of all the clusters.

### Results of multiple comparisons of each HbA1c item in each cluster, and residual analysis in cross-tabulation of HbA1c items

Tables 5, 6 show a comprehensive analysis of HbA1c levels across three distinct clusters.

**TABLE 5:** Results of multiple comparisons for each cluster of the HbA1c values.

HbA1c Values	Cluster 1 *n* = 625		Cluster 2 *n* = 703		Cluster 3 *n* = 582		Significantly different cluster combinations (*p* < 0.05)
	*n* or Median	Ratio (%) or [Interquartile range]	*n* or Median	Ratio (%) or [Interquartile range]	*n* orMedian	Ratio (%) or[Interquartile range]	
Initial HbA1c recorded	6.9	[6.4–7.5]	6.6	[6.3–7.2]	6.8	[6.8–7.8]	1 vs. 2, 2 vs. 3
Initial HbA1c recorded (all groups)							1 vs. 2, 1 vs. 3, 2 vs. 3
1	33	(5.3)	78	(11.1)	91	(15.6)	
2	294	(47)	396	(56.3)	239	(41.1)	
3	209	(33.4)	143	(20.3)	118	(20.3)	
4	89	(14.2)	86	(12.2)	134	(23.0)	
Initial HbA1c recorded (group)							1 vs. 2, 2 vs. 3
<7%	327	(52.3)	474	(67.4)	330	(56.7)	
≥7%	298	(47.7)	229	(32.6)	252	(43.3)	
Final HbA1c recorded	6.9	[6.5–7.6]	6.6	[6.2–7.2]	6.8	[6.2–7.5]	1 vs. 2, 1 vs. 3, 2 vs. 3
Final HbA1c recorded (all groups)							1 vs. 2, 1 vs. 3, 2 vs. 3
1	51	(8.2)	107	(15.2)	96	(16.5)	
2	262	(41.9)	353	(50.2)	242	(41.6)	
3	216	(34.6)	182	(25.9)	141	(24.2)	
4	96	(15.4)	61	(8.7)	103	(17.7)	
Final HbA1c recorded (group)							1 vs. 2, 1 vs. 3, 2 vs. 3
<7%	313	(50.1)	460	(65.4)	338	(58.1)	
≥7%	312	(49.9)	243	(34.6)	244	(41.9)	
Final HbA1c−initial HbA1c	0.1	[−0.5–0.6]	0	[−0.5–0.4]	0	[−0.6–0.3]	1 vs. 3
Final HbA1c−initial HbA1c (all groups)							N. A
−3	4	(0.6)	6	(0.9)	7	(1.2)	
−2	42	(6.7)	37	(5.3)	38	(6.5)	
−1	112	(17.9)	131	(18.6)	98	(16.8)	
0	291	(46.6)	368	(52.3)	320	(55.0)	
+1	142	(22.7)	140	(19.9)	90	(15.5)	
+2	33	(5.3)	20	(2.8)	26	(4.5)	
+3	1	(0.2)	1	(0.1)	3	(0.5)	
Final HbA1c−initial HbA1c (group)							1 vs. 2, 1 vs. 3
Not up	449	(71.8)	542	(77.1)	463	(79.6)	
Up	176	(28.2)	161	(22.9)	119	(20.4)	

“Initial HbA1c recorded” represents the Haemoglobin A1c (HbA1c) level measured at the initial examination in the study period. “Final HbA1c recorded” refers to the HbA1c level at the final examination in the same period. Each HbA1c level was divided into four ranges. Range 1 was HbA1c <6.0%, Range 2 was HbA1c 6.0% to <7.0%, Range 3 was HbA1c 7.0% to <8.0%, and Range 4 was HbA1c ≥8.0% [initial or final HbA1c value recorded (all group): 1, 2, 3, and 4]. Additionally, HbA1c was divided into <7.0% and ≥7.0% [initial or final HbA1c value recorded (group): <7% and ≥7%]. “final HbA1c−initial HbA1c” is the variation of HbA1c ranges calculated by taking the difference between the difference in the number of ranges between the initial HbA1c and final HbA1c recorded and setting it to +1, +2, and +3 as the number of ranges increased, with 0 as no change in range for both ranges; and −1, −2, and −3 as the number of ranges decreased [final HbA1c−initial HbA1c (all group): −3, −2, −1, 0, +1, +2, and +3]. “Not up” represents when the final HbA1c−initial HbA1c (all group) was 0, −1, −2, or −3, and “up” represents when it was +1, +2, or +3 [final HbA1c−initial HbA1c (group): not up and up]. For categorical variables, we used the chi-squared test with a Benjamini and Hochberg (BH) adjustment for multiple comparisons, while the Steel–Dwass test was applied for multiple comparisons of continuous variables between clusters. Statistical significance was set at *p* < 0.05. In the results, “vs.” indicates significant differences between the two clusters (*p* < 0.05). Categorical variables are presented as numbers (%), and continuous variables as medians [interquartile range]. HbA1c, Haemoglobin A1c; “N. A” indicates “not applicable.”

**TABLE 6:** Results of residual analysis for each cluster of HbA1c values.

Values of HbA1c	Cluster 1 *n* = 625			Cluster 2 *n* = 703			Cluster 3 *n* = 582				Adjusted residuals	Adjusted *p*
	Ratio (%)	Adjusted residuals	Adjusted *p*	Ratio (%)	Adjusted residuals	Adjusted *p*	Ratio (%)	Adjusted residuals	Adjusted *p*			
**Initial HbA1c recorded (all groups)**											>0	<0.05
1	5.3	−5.2	<0.001	11.1	0.6	0.573	15.6	4.8	<0.001		>0	<0.1
2	47.0	−1.0	0.360	56.3	5.1	<0.001	41.1	−4.4	<0.001			≥0.1
3	33.4	6.3	<0.001	20.3	−3.3	0.001	20.3	−2.9	0.005		<0	<0.1
4	14.2	−1.6	0.130	12.2	−3.6	0.001	23.0	5.4	<0.001		<0	<0.05
**Initial HbA1c recorded (group)**												
<7%	52.3	−4.3	<0.001	67.4	5.6	<0.001	56.7	−1.5	0.139			
≥7%	47.7	4.3	<0.001	32.6	−5.6	<0.001	43.3	1.5	0.139			
**Final HbA1c recorded (all groups)**												
1	8.2	−4.6	<0.001	15.2	1.9	0.079	16.5	2.7	0.013			
2	41.9	−1.8	0.085	50.2	3.6	0.001	41.6	−1.9	0.079			
3	34.6	4.3	<0.001	25.9	−1.7	0.092	24.2	−2.6	0.018			
4	15.4	1.6	0.120	8.7	−4.8	<0.001	17.7	3.4	0.001			
**Final HbA1c recorded (group)**												
<7%	50.1	−5.0	<0.001	65.4	4.9	<0.001	58.1	−0.1	0.957			
≥7%	49.9	5.0	<0.001	34.6	−4.9	<0.001	41.9	0.1	0.957			
**Final HbA1c−initial HbA1c (all groups)**												
−3	0.6	−0.8	0.695	0.9	−0.1	0.942	1.2	1.0	0.695			
−2	6.7	0.8	0.695	5.3	−1.2	0.605	6.5	0.5	0.735			
−1	17.9	0.1	0.958	18.6	0.7	0.695	16.8	−0.8	0.695			
0	46.6	−2.9	0.044	52.4	0.7	0.695	55.0	2.2	0.130			
+1	22.7	2.5	0.088	19.9	0.4	0.787	15.5	−2.9	0.044			
+2	5.3	1.8	0.280	2.8	−2.2	0.130	4.5	0.5	0.735			
+3	0.2	−0.6	0.714	0.1	−0.8	0.695	0.5	1.4	0.453			
**Final HbA1c−initial HbA1c (group)**												
Not up	71.8	−3.1	0.007	77.1	0.8	0.447	79.6	2.3	0.030			
Up	28.2	3.1	0.007	22.9	−0.8	0.447	20.5	−2.3	0.030			

“Initial HbA1c recorded” represents the Haemoglobin A1c (HbA1c) level measured at the initial examination in the study period. “Final HbA1c recorded” refers to the HbA1c level at the final examination in the same period. Each HbA1c level was divided into four ranges. Range 1 was HbA1c <6.0%, Range 2 was HbA1c 6.0% to <7.0%, Range 3 was HbA1c 7.0% to <8.0%, and Range 4 was HbA1c ≥8.0% [initial or final HbA1c value recorded (all group): 1, 2, 3, and 4]. Additionally, HbA1c was divided into <7.0% and ≥7.0% [initial or final HbA1c value recorded (group): <7% and ≥7%]. “final HbA1c−initial HbA1c” is the variation of HbA1c ranges calculated by taking the difference in the number of ranges between the initial HbA1c and final HbA1c recorded and setting it to +1, +2, and +3 as the number of ranges increased, with 0 as no change in ranges for both ranges; and −1, −2, and −3 as the number of ranges decreased [final HbA1c−initial HbA1c (all group): −3, −2, −1, 0, +1, +2, and +3]. “Not up” represents when the final HbA1c−initial HbA1c (all group) was 0, −1, −2, or −3, and “up” represents when it was +1, +2, or +3 [final HbA1c−initial HbA1c (group): not up and up]. Residual analysis was conducted for each cell, with a Benjamini and Hochberg (BH) adjustment applied to the *p*-values. Red cells signify adjusted residuals greater than 0 and adjusted *p*-values less than 0.05, indicating a cell frequency significantly higher than expected. Light red cells, showing adjusted residuals greater than 0 and adjusted *p*-values less than 0.1, suggest a trend towards higher frequencies, but not significantly so. Conversely, blue cells, with adjusted residuals less than 0 and adjusted *p*-values less than 0.05, indicate a significantly lower frequency, while light blue cells (adjusted residuals less than 0 and adjusted *p*-values less than 0.1) imply a trend towards lower frequencies, although not significantly. HbA1c, Haemoglobin A1c.

Table 5 details the results of multiple comparisons of HbA1c for each cluster. The Steel–Dwass test revealed significant differences in initial HbA1c levels, with Cluster 1 showing the highest levels of all the clusters. Additionally, the chi-squared test with BH adjustment revealed that Cluster 1 had the highest proportion of patients with HbA1c levels greater than or equal to 7%. This pattern was also observed for the final HbA1c measurements, with Cluster 1 again showing the highest levels of all the clusters; however, when considering the change in HbA1c from the initial to the final measurement, denoted as final HbA1c–initial HbA1c, Cluster 1 showed a significant increase compared to Cluster 3. Furthermore, the chi-squared test with BH adjustment showed that Cluster 1 also had the highest proportion of patients with an increase in HbA1c ranges, indicated by “up”.

Table 6 displays the results of the residual test using BH adjustment for the cross-tabulation of each HbA1c item and cluster. In this analysis, Cluster 1 consistently had more patients with HbA1c levels ≥7% than those at the expected frequency, both at the initial and final measurements. When considering the change in HbA1c over time, Cluster 1 had a higher proportion of patients with “up” than expected.

These results highlight that Cluster 1, which is characterised by the patient background depicted on the map in Figure 2, is the group in which the therapeutic efficacy of diabetes over time was the lowest of all the clusters.

## Discussion

In this study, we utilised reports on a leftover drug adjustment protocol from pharmacies to understand the status of outpatient diabetes medication compliance and examined patient characteristics based on basic patient traits, including the presence of any leftover diabetes medications. Furthermore, we classified the patients into three clusters and identified patient groups with low treatment effects and patient characteristics. Patients were classified into three clusters using K-means cluster analysis based on the positional relationships of each patient obtained from each category of patient characteristics based on MCA.

The patient characteristics that are distinctive to Cluster 1 obtained from the map results were DM drug counts (≥3), DM drug prescription duration (≥3 years), Days of DM drug prescription (≥60 d), age (65–74 years), and DM leftover drugs (+). When compared to the other clusters, Cluster 1 may be a patient group with long prescription days and long treatment periods, multiple anti-diabetic drugs used, a majority of patients aged 55–64 years and 65–74 years (pre-old), and several patients with leftover anti-diabetic drugs. Furthermore, Cluster 1 had the highest number of patients with worsening HbA1c levels compared with other clusters, which is an indicator of treatment efficacy.

The patient characteristics that are distinctive to Cluster 2 were age (75–89 years and ≥90 years), all drug counts (≥5), DM drug counts (2), and DM drug prescription duration (1–2 years and 2–3 years). When compared with other clusters, Cluster 2 may be an intermediate group of patients, with several patients being >75 years old, polypharmacy (≥5), and treatment duration being neither long nor short-term. Furthermore, Cluster 2 showed an intermediate HbA1c trend among the three clusters.

The patient characteristics that are distinctive to Cluster 3 were age (<45 years and 45–54 years), all drug counts (<5), DM drug counts (1), Days of DM drug prescription (<30 d and 30–59 d), DM drug prescription duration (<1 year), and leftover DM drugs (−). Compared with the other clusters, Cluster 3 primarily consists of younger patients, those with no polypharmacy, and those with the fewest prescribed diabetes medications. Additionally, this group was characterised by a shorter treatment duration (<1 year) and a lower incidence of leftover anti-diabetic drugs. Furthermore, Cluster 3 had more patients with initial HbA1c 1 and final HbA1c and markedly fewer patients with elevated HbA1c levels than the other clusters.

Thus, the patient characteristics of Cluster 1, in which HbA1c was constantly higher than the reference value during the observation period and in which patients with worsening HbA1c were the most common, included the weakening of the hypoglycaemic effect, leading to a decrease in treatment efficacy, which is a complex predictive factor. Considering that it has been suggested to be achievable, it may be a target for pharmacists to intervene proactively.

This study holds clinical significance. It allowed us to understand the characteristics of patients with diabetes who would benefit from pharmacist intervention, using traits that can be collected in pharmacies.

The relationship between sex and medication adherence in type 2 diabetes is controversial [10, 11, 31–34]. In our study, men and women were located near the origin on the map, and the results of multiple comparisons showed no bias in the ratio of men to women in any cluster. Therefore, in this study, sex was not found to have a direct effect on treatment efficacy. However, there are reports that men have poorer medication adherence [11], and that men in their 40s often discontinue treatment, resulting in low medication adherence [31]. Although both sexes were plotted at the origin in our results, men were plotted at the boundary of the probability ellipses of Clusters 1 and 2, whereas women were included only in Cluster 2. Therefore, if a similar MCA were to be drawn separately for men and women, there may be differences observed in the patient background related to the decrease in the effectiveness of diabetes treatment for each gender. This needs to be considered in future studies.

Studies have reported varying conclusions about the relationship between age and medication compliance in type 2 diabetes [9–11, 31, 33]. A U.S. study concluded that individuals aged 65–74 (pre-old) demonstrated better medication adherence than those aged 45–64. Furthermore, those aged ≥75 showed better medication adherence than the pre-old group [9]. A Japanese study reported better compliance in patients aged 50 to <60 years [11]. In our research, those <55 years were included in Cluster 3, while those 55–64 years moved to the boundary between Clusters 1 and 2. Those aged 65–74 shifted to Cluster 1, and those over 75 moved to Cluster 2, which displayed moderate treatment efficacy and fewer leftover drugs than the other clusters. This shift may be due to an increased reliance on management methods other than self-management with age, such as medication management by family or home visiting services. The residual analysis showed a higher prevalence in the 55–64 and 65–74 age groups in Cluster 1 than in the other age groups. This suggests that patients in these age groups may be less capable of self-management than they or their healthcare providers perceive, or that their treatment may be less effective than expected. Healthcare providers need to intervene more with patients aged 55–64 and 65–74, especially those in the 65–74 age group who are not responding well to treatment.

In a 2017 Japanese study, 43.4% of patients were prescribed one anti-diabetic drug, 31.9% were prescribed two drugs, and 24.5% were prescribed more than three drugs [35]. In this study, the proportion of patients taking three or more medications was slightly higher at 29.4%, aligning with the national distribution, suggesting the potential for generalisability of this study. In Cluster 1, 62.6% of patients were prescribed three or more drugs, which was significantly higher than the national prescription status of anti-diabetic drugs in Japan. As the number of prescribed anti-diabetic drugs increased, the DM drug counts item moved from Cluster 3 to Cluster 2, and then to Cluster 1. The DM drug counts (≥3) was closely positioned to DM drugs (+), both characteristics of patients in Cluster 1 with a poor treatment course. Monotherapy with anti-diabetic drugs has been reported to result in higher treatment adherence than multidrug therapy. Patients switching from multidrug therapy to a single combination tablet have shown improved adherence [36, 37]. Our results support these findings, indicating that in terms of the number of drugs used, patients in Cluster 1 are a group that pharmacists, in collaboration with physicians, should actively intervene on. Therefore, to improve adherence and enhance therapeutic efficacy, pharmacists need to actively monitor the changes in blood glucose and HbA1c levels and intervene, in collaboration with physicians, in reducing the number of antidiabetic prescriptions according to the patient’s condition. This could include strategies such as using combination tablets and changing the drug rather than adding other medications, etc. Even for patients currently on monotherapy, it is necessary to verify whether the treatment strategy is not to increase the number of prescribed drugs.

According to a Japanese survey, more than 60% of patients were prescribed anti-diabetic drugs for up to 30 days, with 90% receiving a prescription for up to 60 days. However, in the target patient group of this study, only 33.7% of patients had prescriptions for less than 60 days. In Cluster 1, 93.6% of patients had a prescription period of 60 days or more. On the map, each item of Days of DM drug prescription moved from Cluster 3 to Cluster 2 and from Cluster 2 to Cluster 1 as the number of prescription days increased, in line with the increase in prescription duration, the DM drug count, and the presence or absence of leftover DM drugs (Figure 2). These results suggest that the longer the number of prescription days for anti-diabetic drugs, that is, the longer the interval between visits to the physician, the lower the therapeutic effect. Therefore, it is expected that the therapeutic effect can be improved by recommending a shortening of the examination interval for patients with long-term prescriptions at one time or for patients who have not achieved effective treatment results. Furthermore, when pharmacists check for leftover drugs according to the protocol, it is necessary to comprehensively consider these issues at the same time and improve the effect of the patient’s diabetes treatment.

Previous studies have shown that medication compliance decreases as the duration of anti-diabetic drug prescriptions increases [38]. The median prescription duration for Clusters 3, 2, and 1 was 0.3, 2.1, and 4.7 years, respectively. As the prescription duration increased from less than 1 year to 3 years or more, patients moved from Cluster 3 to Cluster 1. The duration of DM drug prescriptions was closely related to the count of DM drugs and the presence or absence of leftover DM drugs. The majority of patients in Cluster 1 (85.6%) had been prescribed the drug for more than 3 years, significantly longer than that for patients in other clusters. In Cluster 2, 82.3% of patients had been prescribed the drug for more than 1 year but less than 3 years, and in Cluster 3, 95.9% of patients had been prescribed the drug for less than 1 year. These findings suggest that a prolonged treatment period may reduce the therapeutic effect, necessitating additional interventions. The duration of DM drug prescriptions was also related to age, with younger patients (less than 45 years) more likely to be in Cluster 3. This is because younger patients with shorter disease duration often have more stringent treatment goals [39], leading to a more pronounced hypoglycaemic effect. Therefore, we anticipate that Cluster 3 patients may show decreased medication adherence with increasing years of treatment, suggesting the need for careful monitoring of treatment progression in younger diabetic patients.

The percentages of leftover anti-diabetic drugs were 41.6%, 15.4%, and 3.4% in Clusters 1, 2, and 3, respectively. A 10% change in medication compliance can alter HbA1c levels by 0.15% [40, 41]. It has been demonstrated that pharmacist intervention improves patient adherence to treatment, resulting in lower blood sugar levels [42–45]. In contrast, the target patients in this study underwent pharmacist intervention according to the leftover drug adjustment protocol. The average medication compliance rate was 98.5% for all target patients and remained high at 92.5% even for patients with leftover medication (data not shown). Despite this effective adjustment of leftover drugs, 28.2% of patients in Cluster 1 experienced a decrease in treatment efficacy. This implies that unidentified patients with leftover drugs, or other factors included in cluster 1, may have reduced treatment efficacy.

Accordingly, it will be necessary to implement additional holistic interventions that specifically target patients with low medication adherence. Factors other than the presence of leftover drugs in Cluster 1 could serve as indicators for identifying patients with potential leftover drugs or as intervention points for those whose treatment effects do not improve despite medication correction. These interventions may include reducing the number of drugs, shortening prescription duration, re-selecting therapeutic drugs, and increasing patient awareness by explaining the rationale for the drug regimen. When correcting leftover drugs using the adjustment protocol, consideration of these measures will be essential.

This study has some limitations. First, this was a retrospective, single-centre study. Therefore, the results should be interpreted with caution as they may limit the generalisability of the study. Second, leftover drug adjustment includes patients whose leftover drugs were adjusted according to the leftover drug adjustment protocol. For this reason, we could not include patients who had leftover drugs, but did not request leftover drug adjustment at the pharmacy or patients whose leftover drugs had already been adjusted by their physician at the time of prescription. Third, changes in each patient’s anti-diabetic drugs, anti-diabetic drug dose, or target HbA1c levels were not considered. Fourth, patients prescribed insulin injections or GLP-1 analogue injections were excluded, so the effect on patients receiving these treatments was not considered. These factors may have influenced the results of this study.

In summary, this study has identified the characteristics of patients with low treatment response despite pharmacist intervention using a leftover drug adjustment protocol and patients who were missed by checks under the current protocol and require intervention. Patients aged 65–74 years (pre-old), displayed an increase in the number of anti-diabetic drugs (DM drugs count ≥3), an increase in the years of prescription for anti-diabetic drugs (DM drug prescription duration ≥3 years), and prolonged days of anti-diabetic drug prescription (days of DM drug prescription ≥60 d); in addition leftover anti-diabetic drugs were strongly associated with a decrease in the therapeutic efficacy of anti-diabetic drugs, and these complex factors reduced the therapeutic efficacy. One of the goals of the leftover drug adjustment protocol is to reduce the number of leftover drugs. In the future, it would be advisable to further expand this protocol for protocol-based pharmacotherapy management and to conduct prospective clinical research where pharmacists can intervene in a more complex manner based on the patient factors identified in this study.

## Conclusion

Using a report on the leftover drug adjustment protocol, we clarified the patient characteristics that affected the course of diabetes treatment. These included DM drug counts (≥3), DM drug prescription duration (≥3 years), age (65–74 years), and DM drugs leftover (+). Therefore, it is essential for all healthcare professionals involved in the pharmacological treatment of patients to actively intervene from a comprehensive standpoint, especially when treating those patients exhibiting these characteristics. This intervention includes measures, such as reducing the number of medications, shortening the prescription duration, and explaining the theoretical rationale behind medication regimens to increase patient awareness. These strategies are crucial to our approach to patient care. We anticipate that through these interventions, we can identify those who are taking medication irresponsibly, are not responding well to therapy, or both. This could substantially improve the efficacy of their anti-diabetic care.

## Data Availability

The original contributions presented in the study are included in the article/supplementary material, further inquiries can be directed to the corresponding author.

## Footnotes

1
https://www.R-project.org/

## Abbreviations

BH, Benjamini and Hochberg; DM, Diabetes mellitus; HbA1c, Haemoglobin A1c; MCA, Multiple correspondence analysis.
